# Chronic inflammation following hernia repair and cancer risk: A nationwide study

**DOI:** 10.1016/j.sopen.2025.06.004

**Published:** 2025-06-25

**Authors:** Malene Broholm, Ismail Gögenur, Lau Caspar Thygesen, Frederik Helgstrand

**Affiliations:** a Center for Surgical Science, Department of Surgery, Zealand University Hospital, Koege, Denmark; b Department of Infectious Disease, Copenhagen University Hospital, Hvidovre, Denmark; c National Institute of Public Health, University of Southern Denmark, Copenhagen, Denmark; d Institute for Clinical Medicine, Copenhagen University, Denmark

**Keywords:** Abdominal wall hernia, Inguinal hernia, Cancer, Readmissions, Complications, Mesh, Inflammation

## Abstract

**Purpose:**

Implantation of mesh in patients undergoing hernia surgery or prolonged inflammation due to postoperative complication may be associated with increased risk of cancer.

We aim to test whether implementation of mesh or complicated postoperative course with readmittance was associated with increased risk of cancer.

**Methods:**

This register-based nationwide observational cohort study included 48,392 and 127,756 patients undergoing ventral and inguinal hernia surgery, respectively, during 1996–2004, with follow-up until Dec. 2014. In total, 16,909 patients undergoing ventral hernia repair with mesh, and 31,483 undergoing sutured repair, as well as 106,342 patients undergoing inguinal hernia repair with mesh and 21,414 undergoing sutured repair were included. Patients were matched with a reference cohort from the general Danish population and were followed in the Danish Cancer Registry.

**Results:**

For ventral hernia surgery, there was a significant association between mesh repair and risk of cancers, [(vs general population), HR 1.09 (95%CI, 1.00–1.18)]. Thirty-day readmission after mesh and sutured repair was associated with developing cancer [HR 1.15 (0.99–1.34) and 1.14 (1.00–1.31), respectively]. However, the association for suture repair (HR 1.14) did not reach statistical significance (*p* = 0.055). For inguinal hernia surgery, mesh repair was not associated with increased risk of cancer [(vs. general population), HR 1.00 (95%CI, 0.97–1.02)]. For both mesh and sutured repair, there was an increased risk for developing cancer after 30-day readmission, [HR 1.21 (1.12–1.31) and 1.24 (1.07–1.43), respectively].

**Conclusion:**

Patients undergoing ventral hernia repair with mesh and patients readmitted after inguinal or ventral hernia repair may have higher risk of developing cancer than the general population.

These exploratory findings do not establish causality, but the association warrants further investigations in other populations.

## Introduction

The association between inflammation and malignant disease is well known [[1], [2], [3], [4]]. A characteristic of inflammation is the ability to enable most, if not all, of the core cellular and molecular capabilities required in tumorigenesis [2,5]. An increasing number of studies have suggested an association between the inflammation related to cancer surgery and progression of micrometastasis [6,7]. Moreover, several studies have shown clear associations between a complicated postoperative course after cancer surgery and poor cancer prognosis [8]. However, the association between repetitive surgically-induced acute inflammation or chronic inflammation after surgery and the development of cancer is not clear.

Many surgical procedures for benign diseases involve the implantation of foreign body material that induces local chronic inflammation [9]. Additionally, many of these patients would be undergoing re-operation due to a complication that, in theory, may result in an even more intense inflammation and subsequently in a chronic inflammatory state [10]. The impact of chronic inflammation or complication to benign surgery with or without foreign body material on the development of cancer remains to be investigated in a national population study.

Inguinal and ventral hernia repair are among the most frequent performed benign surgical procedures, with the condition affecting millions of patients worldwide each year [11,12]. With modern operating techniques, the use of foreign body material such as the synthetic meshes has been widely accepted as the gold standard in the treatment of inguinal and abdominal wall hernias. However, based on the nationwide data, 1–6 % of patients have undergone re-operation for complications after ventral hernia repair within the first month, and five years after an incisional hernia repair, 4–6 % have been re-operated for a mesh-related complication and 12 % for a recurrence [[13], [14], [15]]. Our pre-specified null hypotheses was that there were no associations between the risk of developing a cancer either after undergoing hernia surgery with or without mesh reinforcement or after being readmitted after hernia surgery, compared with that in the general population. Based on national data, we aimed to explore the association between surgery for inguinal and abdominal wall hernias and complications after these procedures and the subsequent risk of developing cancer.

## Methods

We conducted a nationwide registry-based observational study using different national Danish registries. The study was approved by the Danish Data Protection Agency (number 2015-57-0008). Since the study is register-based without information on human tissue, according to Danish law, approval from a Research Ethical Committee was not necessary. The manuscript and study results have been reported according to the STROBE statement [16].

### Database registry and cohort definitions

In Denmark, all residents are assigned a unique personal identification number (CPR number) at birth or upon immigration. This CPR number is used in all contacts in Danish healthcare system enabling accurate and complete individual-level linkage of data across all national health and administrative registers, from birth until death or emigration. Registries are mandatory and continuously updated.

Patients eligible for inclusion were all adults (≥18 years) patients who underwent inguinal or ventral hernia suture or mesh repair between 1996 and 2014 registered in the Danish National Patient Register (*n* = 200,679 persons). The Danish National Patient Register contains mandatory reporting on all hospital admissions, including diagnoses and surgical procedures in Denmark since 1977 [17]. Since 1995, the register covers both inpatients and outpatients from all public and private hospitals [17]. We excluded patients with the other hernia surgeries before the index surgery (*n* = 2925), patients admitted before 1996 (*n* = 71) and with missing date of surgery (*n* = 172) ending with 197,511 patients. We stratified the inguinal and ventral hernia repairs into mesh vs. suture and laparoscopic vs. open surgery. Ventral hernia repairs were sub-divided into primary hernias (epigastric and umbilical) and incisional hernias.

A reference cohort from the general Danish population was created and matched 1:1 based on age, sex, and calendar time using the Civil Registration System. The index date for the reference individual was defined as the date of the matched patient's hernia surgery. This index date was not associated with a healthcare event for the control individual, but was assigned to ensure temporal alignment and equal follow-up time between patients and their matched reference individuals. The reference cohort was based on data from the Civil Registration System, which includes all individuals with a permanent residence in Denmark [18].

All patients and reference cohort were excluded if not with residency in Denmark on index date (*n* = 561 patients and references excluded). Furthermore, patients and references with cancer (excluded non-melanoma skin cancer) before index date were excluded (*n* = 23,563), mortality at index date (*n* = 13) or emigration on index date (n = 1). We also removed patients and reference cohort without matching comparison person resulting in a patient and reference cohorts of 174,531 persons each whereof 127,756 had inguinal hernias and 48,392 had ventral hernia (1617 had both hernias surgeries).

All individuals in the intervention and reference cohort were followed in the Danish Cancer Registry [19], which is a nationwide register established in 1942 with data available from 1943. The register records all patients diagnosed with a cancer in Denmark with a validity and completeness of 95–98 % [19].

### Data extraction

We included information on several confounders at time of hernia repair (intervention cohort) or index date (reference cohort): age, sex, Charlson comorbidity index (CCI), chronic obstructive pulmonary disease (COPD), educational level, affiliation with the labour market, a job with heavy work and calendar time. Information on confounders was obtained from the Danish Civil Registration System [18], the Danish National Patient Register [17], the Population's Education Register [20], and the Integrated Database for Labour Market Research [21]. All confounders were described using means, standard deviation, and proportions.

All patients with hernia and the reference group were followed from the date of hernia repair or index date until first cancer diagnosis (excluding non-melanoma skin cancer), emigration, disappearance, death, or end of the study period (31 December 2014). For the reference group, the follow-up time was censored at the time of a subsequent hernia repair operation after the initial matching. Individuals with a cancer diagnosis (excluding non-melanoma skin cancer) before hernia surgery or index date were excluded.

### Statistical analyses

As descriptive measures, we performed Kaplan-Meier survival analysis of the patients with hernia undergoing mesh repair and the matched general population. Cox regression analyses were performed, adjusting for the confounding factors, and reported as hazard ratios (HR) and 95 % confidence intervals (95%CI). The proportional hazard assumption was visually evaluated of survival curves indicating no violations. *P*-values <0.05 were considered statistically significant. Since loss to follow-up and missing data are minimal in Danish registries no corrections for missing data or loss to follow-up were performed. The main analysis was the comparison of patients undergoing mesh surgery with the general population. We performed pre-planned supplementary analyses to evaluate the robustness of the main results: a) comparison of patients who underwent mesh repair to those who underwent sutured hernia repair, b) comparison of patients undergoing mesh surgery with complications registered as readmission within 30 days of surgery versus patients with mesh repair without readmission within 30 days, c) comparison of cancer development in patients with reference population among those without any other comorbidities (CCI = 0), d) comparison with patients with reference population among those with comorbidities (CCI = 2+), and e) cancer-specific analyses.

## Results

### Ventral hernia

Overall, 16,909 patients underwent a ventral hernia repair with mesh, and 31,483 underwent sutured repair. There were more males in the mesh (56 %) and sutured repair (58 %) groups. A total of 35 % of the patients from the mesh group were operated using a laparoscopic technique. Readmittance was seen in 12 % of the patients within 30 days in the mesh repair group and 8 % in the sutured repair group (Table 1).

**Table 1:** Baseline descriptive of ventral (primary and incisional) and inguinal hernia repairs with and without mesh and the matched general population. Numbers and percentages if nothing else noted.

	Ventral hernia repairs with mesh vs matched population		Ventral hernia repairs with suture vs. matched population		Inguinal hernia repairs with mesh vs matched population		Inguinal hernia repairs with suture vs matched population	
	Mesh	Matched	Sutured	Matched	Mesh	Matched	Sutured	Matched
N	16,909	16,909	31,483	31,483	106,342	106,342	21,414	21,414
Follow-up, mean (min–max)	6.2 (0–19)	6.4 (0–19)	8.2 (5.4)	8.4 (0–19)	8.0 (0–19)	7.7 (0–19)	11.7 (0–19)	11.3 (0–19)
Age, mean (SD)	54.3 (13.6)	54.3 (13.6)	49.9 (14.7)	49.9 (14.7)	56.4 (15.7)	56.4 (15.7)	54.0 (17.8)	54.0 (17.8)
Age, n (%)								
18–39	2589 (15)	2589 (15)	8402 (27)	8402 (27)	16,581 (16)	16,581 (16)	4973 (23)	4973 (23)
40–59	8094 (48)	8094 (48)	14,749 (47)	14,749 (47)	41,920 (39)	41,920 (39)	7659 (36)	7659 (36)
60–79	5758 (34)	5758 (34)	7493 (24)	7493 (24)	41,568 (39)	41,568 (39)	7382 (34)	7382 (34)
80+	468 (3)	468 (3)	839 (3)	839 (3)	6273 (6)	6273 (6)	1400 (7)	1400 (7)
Males, n (%)	9528 (56)	9528 (56)	18,402 (58)	18,402 (58)	98,736 (93)	98,736 (93)	18,687 (87)	18,687 (87)
Calendar period								
1996–2000	2010 (12)	2010 (12)	9102 (29)	9102 (29)	25,445 (24)	25,445 (24)	14,971 (70)	14,971 (70)
2001–2005	3887 (23)	3887 (23)	8438 (27)	8438 (27)	32,862 (31)	32,862 (31)	4463 (21)	4463 (21)
2006–2010	5857 (35)	5857 (35)	7168 (23)	7168 (23)	27,798 (26)	27,798 (26)	1302 (6)	1302 (6)
2011–2014	5155 (30)	5155 (30)	6775 (22)	6775 (22)	20,237 (19)	20,237 (19)	678 (3)	678 (3)

Surgical information								
Operation type								
Laparoscopic	5917 (35)	–	0 (0)	–	13,972 (13)	–	0 (0)	–
Open	10,992 (65)	–	31,483 (100)	–	92,370 (87)	–	21,414 (100)	–
Operations before hernia repair	13,439 (79)	9155 (54)	19,763 (63)	14,566 (46)	54,803 (52)	47,619 (45)	6207 (29)	5162 (24)
Operations after repair (>30 days)	10,749 (64)	8271 (49)	21,327 (67)	17,358 (55)	68,490 (64)	59,505 (56)	16,371 (76)	14,714 (69)

Socio-economic								
Educational level								
Elementary school	6282 (37)	5161 (31)	10,734 (34)	9229 (29)	32,599 (31)	32,978 (31)	6962 (33)	7223 (34)
High school	548 (3)	658 (4)	1259 (4)	1601 (5)	3631 (3)	4466 (4)	861 (4)	1013 (5)
Vocational education	6432 (38)	6256 (37)	11,748 (37)	11,427 (36)	42,807 (40)	40,074 (38)	7776 (36)	7161 (33)
Higher education	2977 (18)	4185 (25)	6289 (20)	7658 (24)	21,117 (20)	22,161 (21)	3503 (16)	3686 (17)
Missing	670 (4)	649 (4)	1453 (5)	1568 (5)	6188 (6)	6663 (6)	2312 (11)	2331 (11)
Labour market affiliation								
Active	8327 (49)	10,023 (59)	18,689 (59)	20,225 (64)	60,044 (56)	57,304 (54)	11,802 (55)	11,469 (54)
Unemployed	1915 (11)	1372 (8)	3747 (12)	3308 (11)	6399 (6)	7745 (7)	1691 (8)	1937 (9)
Pensioner	6667 (39)	5514 (33)	9047 (29)	7950 (25)	39,899 (38)	41,293 (39)	7921 (37)	8008 (37)
Job with heavy work	301 (2)	264 (2)	446 (1)	388 (1)	1221 (1)	1023 (1)	50 (0)	35 (0)

Comorbidity								
Charlson score								
0	11,633 (69)	14,993 (89)	25,229 (80)	28,529 (91)	90,130 (85)	91,463 (86)	18,424 (86)	18,883 (88)
1	3174 (19)	1312 (8)	3843 (12)	1998 (6)	11,096 (10)	9529 (9)	1957 (9)	1667 (8)
2	1251 (7)	404 (2)	1319 (4)	616 (2)	3182 (3)	3288 (3)	657 (3)	563 (3)
3–4	704 (4)	161 (1)	882 (3)	276 (1)	1586 (1)	1690 (2)	309 (1)	250 (1)
5+	147 (1)	39 (0)	210 (1)	64 (0)	348 (0)	372 (0)	67 (0)	51 (0)
COPD	1503 (9)	421 (2)	1838 (6)	625 (2)	4322 (4)	2950 (3)	679 (3)	502 (2)

Abbreviations: HR, hazard ratio; SD, standard deviation; COPD, chronic obstructive pulmonary disease.

**Table 2:** Association between ventral hernia repair and cancer incidence, Denmark, 1996–2014.

Analyses		Cancer cases	Risk time	Persons	IR (1)	Unadj HR (2)	Adj HR (3)
Mesh repair vs. matched population	Mesh repair	1471	104,347.3	16,909	1409.7	1.26 (1.16–1.35)	1.09 (1.00–1.18) (4)
	General population	1215	107,699.7	16,909	1128.2	1.00 (ref)	1.00 (ref)
	Mesh + readmission	192	11,306.3	2041	1698.2	1.44 (1.16–1.78)	1.17 (0.93–1.48)
	General population	151	12,612.6	2041	1197.2	1.00 (ref)	1.00 (ref)
	Mesh - readmission	1279	93,041.0	14,868	1374.7	1.23 (1.14–1.34)	1.08 (0.99–1.17)
	General population	1064	95,087.1	14,868	1119.0	1.00 (ref)	1.00 (ref)
	Mesh repair, Charlson = 0	859	75,464.4	11,633	1138.3	1.15 (1.05–1.26)	1.14 (1.04–1.25)
	General population	1043	97,636.7	14,993	1068.3	1.00 (ref)	1.00 (ref)
	Mesh repair, Charlson ≥2	252	10,461.0	2102	2409.0	1.12 (0.85–1.48)	1.09 (0.82–1.44)
	General population	63	2787.2	604	2260.4	1.00 (ref)	1.00 (ref)
Mesh vs. sutured repair	Mesh repair	1471	104,347.3	16,909	1409.7	1.13 (1.06–1.21)	1.07 (1.00–1.14) (5)
	Sutured repair	2740	256,979.5	31,483	1066.2	1.00 (ref)	1.00 (ref)
30-Day readmission with mesh repair	Readmitted	192	11,306.3	2041	1698.2	1.23 (1.06–1.44)	1.15 (0.99–1.34)
	Not readmitted	1279	93,041.0	14,868	1374.7	1.00 (ref)	1.00 (ref)
30-Day readmission with sutured repair	Readmitted	229	16,644.3	2409	1375.9	1.24 (1.09–1.42)	1.14 (1.00–1.31) (6)
	Not readmitted	2511	240,335.2	29,074	1044.9	1.00 (ref)	1.00 (ref)

(1) Incidence rate per 100,000 person-years.

(2) Hazard ratio from Cox regression model matched on age and sex. Numbers in parentheses are 95 % confidence interval.

(3) Hazard ratio from Cox regression model matched on age and sex and adjusted for Charlson comorbidity index, COPD, educational level, affiliation with the labour market, job with heavy work and calendar time (5 years periods).

(4) p-value = 0.041.

(5) p-value = 0.057.

(6) p-value = 0.055.

NOTE: Test for interaction between mesh hernia and Charlson comorbidity (0, 1, 2, 3–4 and 5+): chi2 = 3.4, df = 4, *p* = 0.50.

A total of 1471 had a cancer diagnosis after their ventral hernia repair with mesh compared with 1215 patients in the general population (Table 2; see Table S1 for cancer subtypes). There was a significant association between ventral hernia repair with mesh (vs. general population) and risk of later cancer with an adjusted HR (Adj HR) 1.09 (95 % CI, 1.00–1.18) (Table 2). In a supplementary analysis, the ventral hernias were subdivided into primary ventral (umbilical or epigastric) and incisional hernias. Although not significant, the association between increased cancer risk for both the umbilical/epigastric repairs [adj HR 1.09 (0.95–1.24)] and the incisional hernia repairs [adj HR 1.09 (0.99–1.20)] compared to the matched general population, persisted (Table S2). A readmission after mesh repair and sutured repair resulted in an increased risk for developing cancer [adj HR 1.15 (0.99–1.34) and adj HR 1.14 (1.00–1.31), respectively] (Table 2). Although not significant, patients with a mesh repair and readmission had a higher adjusted risk for cancer compared with the general population [adj HR 1.17 (0.93–1.48)] than patients with a mesh repair without readmission [adj HR 1.08 (0.99–1.17)] (Table 2). This tendency was also found in the sub-analyses for patients undergoing small primary ventral hernia repairs and those with an incisional hernia repair (Table S2). The tendency towards higher risk for cancer also persisted when the mesh repairs were subdivided into open and laparoscopic repair (Table S3). An increased risk of cancer was also observed in patients with a CCI of 0 undergoing mesh operation compared with the general population [adj. HR 1.14 (1.04–1.25)], but mesh repair and CCI ≥2 was not associated with an increased risk for later cancer [adj. HR 1.09 (0.82–1.44)]. Finally, the cancer incidence tended to be higher in the mesh repair group compared with the sutured group [HR 1.07 (1.00–1.14)]; however, this did not reach statistical significance (*p* = 0.057) (Table 2).

**Fig. 1:**
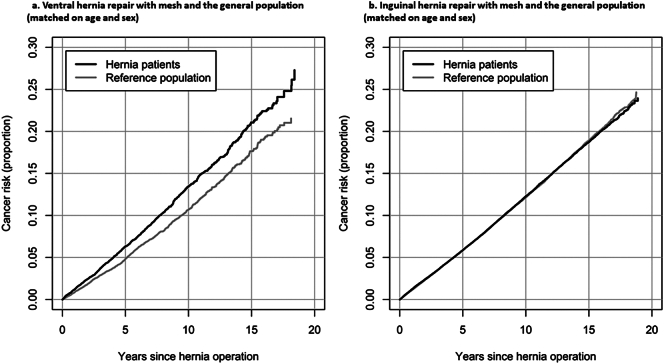
Risk of cancer development.

The cancer-free period after mesh hernia surgery is depicted in Fig. 1a, showing a higher risk for cancer development over time in the ventral mesh group compared with the general population (Log-rank *p*-value<0.0001).

### Inguinal hernia

Overall, 106,342 patients with an inguinal hernia operated with mesh implantation and 21,414 patients operated without mesh implantation were included in the inguinal hernia group (Table 1).

Inguinal hernia repairs were mainly performed on male patients (93 % and 87 % in the mesh and sutured group, respectively). In the mesh group, 13 % of the patients were operated using laparoscopic technique (no patients underwent a laparoscopic sutured repair). A total of 5 % of the patients were readmitted within 30 days in the mesh group and 6 % in the sutured repair group. The corresponding number of patients in the matched general population operated (any operation) within 30 days after inclusion in the cohort were 2 % and 1 % for the mesh and sutured groups, respectively.

**Table 3:** Association between inguinal hernia repair and cancer incidence, Denmark, 1996–2014.

Analyses		Cancer cases	Risk time	Persons	IR (1)	Unadj HR (2)	Adj HR (3)
Mesh repair vs. matched population	Mesh hernia patients	11,262	850,065.7	106,342	1324.8	1.00 (0.97–1.03)	1.00 (0.97–1.02)
	General population	10,788	815,428.4	106,342	1323.0	1.00 (ref)	1.00 (ref)
	Mesh + readmission	666	36,790.4	5843	1810.3	1.14 (1.02–1.27)	1.11 (0.99–1.24)
	General population	612	38,545.3	5843	1587.7	1.00 (ref)	1.00 (ref)
	Mesh - readmission	10,596	813,275.3	100,499	1302.9	0.99 (0.96–1.02)	0.99 (0.97–1.02)
	General population	10,176	776,883.1	100,499	1309.9	1.00 (ref)	1.00 (ref)
	Mesh repair, Charlson = 0	9180	753,729.2	90,130	1217.9	0.98 (0.96–1.01)	0.99 (0.96–1.02)
	General population	8875	731,158.5	91,463	1213.8	1.00 (ref)	1.00 (ref)
	Mesh repair, Charlson ≥2	655	25,900.6	5116	2528.9	1.03 (0.92–1.15)	1.03 (0.92–1.15)
	General population	628	25,562.0	5350	2456.8	1.00 (ref)	1.00 (ref)
Mesh vs. sutured repair	Mesh repair	11,262	850,065.7	106,342	1324.8	0.99 (0.95–1.03)	0.95 (0.91–0.99)
	Sutured repair	3148	249,894.9	21,414	1259.7	1.00 (ref)	1.00 (ref)
30-Day readmission with mesh repair	Readmitted	666	36,790.4	5843	1810.3	1.26 (1.17–1.36)	1.21 (1.12–1.31)
	Not readmitted	10,596	813,275.3	100,499	1302.9	1.00 (ref)	1.00 (ref)
30-Day readmission with sutured repair	Readmitted	190	10,415.7	1231	1824.2	1.29 (1.12–1.50)	1.24 (1.07–1.43)
	Not readmitted	2958	239,479.1	20,183	1235.2	1.00 (ref)	1.00 (ref)

(1) Incidence rate per 100,000 person-years.

(2) Hazard ratio from Cox regression model matched on age and sex. Numbers in parentheses are 95 % confidence interval.

(3) Hazard ratio from Cox regression model matched on age and sex and adjusted for Charlson comorbidity index, COPD, educational level, affiliation with the labour market, job with heavy work and calendar time (5 years periods).

A total of 11,262 patients with a previous inguinal hernia repair with mesh developed cancer compared with 10,788 cases in the general population (Table 3 and S4 for cancer subtypes). Compared with the general population, mesh repair was not associated with increased cancer incidence [adj HR) 1.00 (95 % CI, 0.97–1.02)] (Table 3). Further, the survival was the same for patients undergoing inguinal mesh hernia repair compared with the general population (Fig. 1b). For both the mesh and sutured groups, there was an increased likelihood for developing a cancer among those with a readmission within 30 days after surgery with an adj HR of 1.21 (1.12–1.31) for the mesh group and 1.24 (1.07–1.43) for the sutured group (Table 3). As for the ventral hernias, an insignificant higher adjusted cancer risk was found for patients with a mesh repair and readmission compared with the general population [adj HR = 1.11 (0.99–1.24] than patients with a mesh repair but without readmission (adj HR = 0.99 (0.97–1.02)) (Table 3). There were no associations between hernia surgery and cancer risk among those with and without comorbidities and a slight decreased risk with mesh repair compared to sutured repair [0.95; 0.91–0.99] (Table 3). Readmission after both open [adj HR = 1.18 (1.09–1.29)] and laparoscopic mesh repair [adj HR = 1.44 (1.13–1.83)] significantly increased the risk for later cancer compared with patients who did not get readmitted (Table S3).

### Sensitivity analysis

A sensitivity analysis was conducted to investigate whether the same associations were present for patients undergoing laparoscopic or open inguinal or ventral hernia mesh repair (Table S4), which revealed that the associations were still present in the inguinal hernia group but did not reach statistical significance in the ventral hernia group.

## Discussion

In this nationwide register-based study investigating the risk of developing a cancer in patients undergoing inguinal and ventral hernia surgery, we found no overall association between the placement of a mesh and the development of cancer after abdominal hernia repair, when comparing with the general population. In addition, an increased risk of developing cancers among patients readmitted after both inguinal and ventral hernia surgery was found. Although numerically higher, 30-day readmitted patients with a mesh repair had no statistically significant risk for developing cancer compared with the matched general population. This tendency sustained in the sub-analysis of open and laparoscopic procedures, indicating that post-operative complications may be a driver for later cancer development regardless of the surgical technique utilized.

Recently, a register-based study exhibited that 5 % of the patients undergoing surgery for ventral hernia are re-operated due to mesh complications [14]. Our findings indicate that there may be a need for evaluating of the long-term consequences of mesh implantation or other foreign body implantations on the risk of cancer and a need to explore the mechanism that may underlie our results. We have clearly not established the causal mechanism of the association. Although not comparable to our cohort, other studies have shown that there is a highly complex immune infiltration in patients who have their meshes removed [22]. These immune infiltrations not only represent a macrophage and fibroblast inflammatory response but also involved cytotoxic lymphocytes. It can be hypothesized that the mesh surgery in patients undergoing ventral hernia repair or those undergoing a reoperation in a milieu of surgical stress response after the initial surgery can result in a tolerance state in certain immune active cells. Thus, it may be possible that mesh implantation and/or a complication within 30 days (mainly infectious complication) results in an immunocompromised state that can reduce the immunosurveillance necessary for preventing cancer occurrence systemically. Furthermore, the presence of foreign body material may induce a chronic inflammatory state as known in chronic infections which subsequently can result in an increased risk of developing cancer [[23], [24], [25], [26]]. Patients with hernias have an altered collagen matrix [27], and maybe these patients also have other genetic variations predisposing to cancer. Our study has several strengths. First, we used a nationwide cohort including all patients, who underwent hernia surgery, which was identified independently of the cancer outcome studied. The large size of the cohort minimizes the influence of random variation. Second, we included an age, sex, and calendar time-matched reference population, and we obtained complete follow-up of cancer outcome, death, and emigration for all patients. Furthermore, we retrieved individual-level information on several important confounders. The objective registration minimizes bias from subjective reporting. Finally, since all hernia patients were included, the representativeness of the results is ensured [23].

There were several important limitations in our study, mainly based on the limited clinical information on the patients included. While information regarding the surgical procedure and major comorbidities was available, the influence of unmeasured confounding factors, such as obesity or medical therapies with potential implications for cancer risk could not be ruled out. Additionally, we assumed that readmission after abdominal wall hernia surgery was representing an additional inflammatory state due to infection or re-operation. This assumption was supported by a previous register-based study showing that infectious complications and reoperations are the main reason for readmissions after abdominal wall surgery. Our results may also reflect that we were unable to control for unmeasured confounders that may influence the associations reported and therefore our findings cannot indicate causality. One of these unmeasured confounders with respect to the patients with ventral hernias could be primary surgery for another benign condition, leading to an incisional hernia. Thus, the underlining condition leading to the benign surgery might have been a proxy for a certain risk group. However, we did not observe any difference in lifestyle-related cancers such as lung cancer between the two groups indicating that lifestyle factors have only a minor influence on the results. Another potential limitation is that we conducted many statistical tests thereby increasing the risk of false positive results.

A significant part of the increased cancer risk observed in our study is attributable to prostate cancer. Several factors may contribute to this association, first of all, this is a very common cancer form but chronic postoperative inflammation, may also explain the increased cancer risk. Additionally, patients undergoing surgery are more likely to have increased healthcare interactions, potentially leading to higher detection rates of low grade prostate cancer. While the exact mechanisms remain uncertain, these findings suggest that further research is needed to better understand the link between hernia repair and prostate cancer risk. Further studies are needed to investigate whether the same associations are present in other benign conditions where mesh material is incorporated such as urogenital procedures, orthopedic surgery, stent implantations in vessels, and so forth to establish the associations found in this study. Moreover, detailed immunological studies should be performed to explore the underlying differences in the immune profile of patients undergoing ventral hernia surgery with or without mesh and to specifically monitor the immunological changes related to readmission in order to find possible explanations between the different groups investigated in our study.

In conclusion, we have shown that patients undergoing a ventral hernia mesh repair are significantly at risk for a later cancer diagnosis. Although no association between implantation of mesh and risk of cancer development in patients undergoing inguinal hernia repair was found, all patients having a complicated course with readmission regardless of the type of repair seemed to have an increased risk of cancer. Further studies are required to establish this association and to investigate possible mechanisms in order to develop preventive measures.

The following are the supplementary data related to this article.

Supplementary Table 1 Cancer-specific analyses of ventral mesh hernia patients and cancer incidence, Denmark, 1996–2014. Supplementary Table 1

Supplementary Table 2 Association primary ventral and incisional hernia repair with mesh and cancer incidence compared to matched general population, Denmark, 1996–2014. Supplementary Table 2

Supplementary Table 3 Cancer-specific analyses of inguinal mesh hernia patients and cancer incidence, Denmark, 1996–2014. Supplementary Table 3

Supplementary Table 4 Supplementary analyses comparing open versus laparoscopic mesh hernia repair with readmission stratified by incisional and ventral mesh hernia repair and cancer incidence, Denmark, 1996–2014. Supplementary Table 4

## Code available

Not applicable.

## CRediT authorship contribution statement

**Malene Broholm:** Writing – review & editing, Writing – original draft. **Ismail Gögenur:** Writing – review & editing, Methodology, Formal analysis, Conceptualization. **Lau Caspar Thygesen:** Writing – review & editing, Validation, Methodology, Formal analysis. **Frederik Helgstrand:** Writing – review & editing, Supervision, Resources, Project administration, Methodology.

## Informed consent

All procedures performed in this study were in accordance with the Danish ethical standards and in accordance with the 1964 Helsinki declaration and its later amendments. According to Danish law, studies based solely on data from the Danish national registers do not need approval from the Danish research bioethics committees, as study participants are never contacted, and consent is not required for the use of register information.

## Consent for publication

Study participants are never contacted, and consent is not required for the use of register information. Informed consent for publication from each individual participant was therefore not necessary.

## Ethics approval

Since the study is register-based without information on human tissue, approval from a Research Ethical Committee was not necessary, according to the Danish law.

## Funding

The work received no funding.

## Declaration of competing interest

The authors declare that they have no known competing financial interests or personal relationships that could have appeared to influence the work reported in this paper.

## Data Availability

This study is based on Danish national register data. These data do not belong to the authors but to the Danish Health Data Authority and the authors are not permitted to share them, except in aggregate (e.g. a publication).

## References

[1] Balkwill F., Mantovani A. (2001). Inflammation and cancer: back to Virchow?. Lancet.

[2] Elinav E., Nowarski R., Thaiss C., Jin C., Flavell R.A. (2013). Inflammation-induced cancer: crosstalk between tumours, immune cells and microorganisms. Nat Rev Cancer.

[3] Grivennikov S.I., Greten F.R., Karin M. (2010). Immunity, inflammation, and cancer. Cell.

[4] Coussens L.M., Werb Z. (2002). Inflammation and cancer. Nature.

[5] Hanahan D., Weinberg R.A. (2011). Hallmarks of cancer: the next generation. Cell.

[6] Lee K.T., Jung J.H., Mun G.H., Pyon J.K., Bang S.I., Nam S.J. (2020). Influence of complications following total mastectomy and immediate reconstruction on breast cancer recurrence. Br J Surg.

[7] Wang Y., Qu M., Qiu Z. (2022). Surgical stress and Cancer progression: new findings and future perspectives. Curr Oncol Rep.

[8] Hiller J.G., Perry N.J., Poulogiannis G., Riedel B., Sloan E.K. (2018). Perioperative events influence cancer recurrence risk after surgery. Nat Rev Clin Oncol.

[9] Klinge U., Park J.K., Klosterhalfen B. (2013). The ideal mesh?. Pathobiology.

[10] Kokotovic D., Burcharth J., Helgstrand F., Gögenur I. (2017). Systemic inflammatory response after hernia repair: a systematic review. Langenbecks Arch Surg.

[11] Bay-Nielsen M., Kehlet H., Strand L. (2001). Quality assessment of 26 304 herniorrhaphies in Denmark: a prospective nationwide study. Lancet.

[12] Poulose B.K., Shelton J., Phillips J. (2012). Epidemiology and cost of ventral hernia repair: making the case for hernia research. Hernia.

[13] Helgstrand F., Rosenberg J., Kehlet H., Bisgaard T. (2013). Outcomes after emergency versus elective ventral hernia repair: a prospective nationwide study. World J Surg.

[14] Bisgaard T., Kokotovic D., Helgstrand F. (2017). Recurrence and mesh-related complications after incisional hernia repair-reply. JAMA.

[15] Bay-Nielsen M., Kehlet H. (2008). Anaesthesia and post-operative morbidity after elective groin hernia repair: a nation-wide study. Acta Anaesthesiol Scand.

[16] von Elm E., Altman D.G., Egger M., Pocock S.J., Gøtzsche P.C., Vandenbroucke J.P. (2014). The strengthening the reporting of observational studies in epidemiology (STROBE) statement: guidelines for reporting observational studies. Int J Surg.

[17] Lynge E., Sandegaard J.L., Rebolj M. (2011). The Danish national patient register. Scand J Public Health.

[18] Pedersen C.B. (2011). The Danish civil registration system. Scand J Public Health.

[19] Storm H.H., Michelsen E.V., Clemmensen I.H., Pihl J. (1997). The Danish cancer registry history, content, quality and use. Dan Med Bull.

[20] Jensen V.M., Rasmussen A.W. (2011). Danish education registers. Scand J Public Health.

[21] Baadsgaard M., Quitzau J. (2011). Danish registers on personal income and transfer payments. Scand J Public Health.

[22] Klosterhalfen B., Klinge U. (2013). Retrieval study at 623 human mesh explants made of polypropylene—impact of mesh class and indication for mesh removal on tissue reaction. J Biomed Mater Res B Appl Biomater.

[23] Thygesen L.C., Ersbøll A.K. (2014). When the entire population is the sample: strengths and limitations in register-based epidemiology. Eur J Epidemiol.

[24] Chiba T., Marusawa H., Ushijima T. (2012). Inflammation-associated cancer development in digestive organs: mechanisms and roles for genetic and epigenetic modulation. Gastroenterology.

[25] Ben-Neriah Y., Karin M. (2011). Inflammation meets cancer, with NF-κB as the matchmaker. Nat Immunol.

[26] Kuraishy A., Karin M., Grivennikov S.I. (2011). Tumor promotion via injury- and death-induced inflammation. Immunity.

[27] Henriksen N.A., Yadete D.H., Sorensen L.T., Ågren M.S., Jorgensen L.N. (2011). Connective tissue alteration in abdominal wall hernia. Br J Surg.

