# Supplementary material for: Chronic inflammation following hernia repair and cancer risk: A nationwide study
Source: Surg Open Sci. 2025 Jun 25;27:88–93. doi: 10.1016/j.sopen.2025.06.004 (PMC12301763; doi:10.1016/j.sopen.2025.06.004)
Supplement: Supplementary Table 2 — Association primary ventral and incisional hernia repair with mesh and cancer incidence compared to matched general population, Denmark, 1996–2014. [file mmc2.docx]

**Supplementary Table 2.**

**Association primary ventral and incisional hernia repair with mesh and cancer incidence compared to matched general population, Denmark, 1996-2014**

| **Analyses** | |  | **Cancer cases** | **Risk time** | **Persons** | **IR (1)** |  | **Adj HR (2)** |
| --- | --- | --- | --- | --- | --- | --- | --- | --- |
|  |  |  |  |  |  |  |  |  |
| **Primary ventral hernia repairs** | |  |  |  |  |  |  |  |
|  | With mesh |  | 472 | 39,228.1 | 7,710 | 1,203.2 |  | 1.09 (0.95-1.24) |
|  | General population |  | 400 | 39,631.0 | 7,710 | 1,009.3 |  | 1.00 (ref) |
|  |  |  |  |  |  |  |  |  |
|  | 30-day readmission |  | 51 | 3,230.1 | 737 | 1,578.9 |  | 1.24 (0.92-1.66) |
|  | Not readmitted |  | 421 | 35,998.0 | 6,973 | 1,169.5 |  | 1.00 (ref) |
|  |  |  |  |  |  |  |  |  |
|  | With mesh + 30-day readmission |  | 51 | 3,230.1 | 737 | 1,578.9 |  | 1.23 (0.78-1.94) |
|  | General population |  | 39 | 3,578.8 | 737 | 1,089.8 |  | 1.00 (ref) |
|  |  |  |  |  |  |  |  |  |
|  | With mesh – 30 day readmission |  | 421 | 35,998.0 | 6,973 | 1,169.5 |  | 1.07 (0.93-1.24) |
|  | General population |  | 361 | 36,052.2 | 6,973 | 1,001.3 |  | 1.00 (ref) |
|  |  |  |  |  |  |  |  |  |
|  |  |  |  |  |  |  |  |  |
| **Incisional hernia repair** | |  |  |  |  |  |  |  |
|  | Incisional hernia patients with mesh |  | 1,005 | 65,775.4 | 9,326 | 1,527.9 |  | 1.09 (0.99-1.20) |
|  | General population |  | 823 | 68,726.9 | 7,326 | 1,197.5 |  | 1.00 (ref) |
|  |  |  |  |  |  |  |  |  |
|  | 30-day readmission |  | 142 | 8,138.6 | 1,317 | 1,744.8 |  | 1.13 (0.94-1.35) |
|  | Not readmitted |  | 863 | 57,636.8 | 8,009 | 1,497.3 |  | 1.00 (ref) |
|  |  |  |  |  |  |  |  |  |
|  | With mesh + 30-day readmission |  | 142 | 8,138.6 | 1,317 | 1,744.8 |  | 1.15 (0.87-1.51) |
|  | General population |  | 112 | 9,103.5 | 1,317 | 1,230.3 |  | 1.00 (ref) |
|  |  |  |  |  |  |  |  |  |
|  | With mesh – 30-day readmission |  | 863 | 57,636.8 | 8,009 | 1,497.3 |  | 1.08 (0.97-1.20) |
|  | General population |  | 711 | 59,623.4 | 8,009 | 1,192.5 |  | 1.00 (ref) |
|  |  |  |  |  |  |  |  |  |

(1) Incidence rate per 100,000 person-years.

(2) Hazard ratio from Cox regression model matched on age and sex and adjusted for Charlson comorbidity index, COPD, educational level, affiliation with the labour market, job with heavy work and calendar time (5 years periods).
