# Supplementary material for: Chronic inflammation following hernia repair and cancer risk: A nationwide study
Source: Surg Open Sci. 2025 Jun 25;27:88–93. doi: 10.1016/j.sopen.2025.06.004 (PMC12301763; doi:10.1016/j.sopen.2025.06.004)
Supplement: Supplementary Table 3 — Cancer-specific analyses of inguinal mesh hernia patients and cancer incidence, Denmark, 1996–2014. [file mmc3.docx]

**Supplementary Table 3.**

**Cancer-specific analyses of inguinal mesh hernia patients and cancer incidence, Denmark, 1996-2014**

| **Cancer site** | **Cancer cases** | |  | **Adj HR (1)** |
| --- | --- | --- | --- | --- |
|  | **Mesh patients** | **General population** |  |  |
| Any cancer (C00-C96) | 11,262 | 10,788 |  | 1.00 (0.97-1.02) |
| Buccal cavity and pharynx (C00-C14) | 268 | 277 |  | 0.93 (0.79-1.10) |
| Lip (C00) | 17 | 27 |  | 0.61 (0.33-1.12) |
| Tongue (C01-C02) | 38 | 43 |  | 0.83 (0.54-1.29) |
| Mouth (C03-C06) | 66 | 76 |  | 0.85 (0.61-1.19) |
| Pharynx (C10-C13) | 68 | 61 |  | 1.08 (0.76-1.52) |
| Digestive organs and peritoneum (C15-C26) | 2,593 | 2,761 |  | 0.90 (0.85-0.95) |
| Esophagus (C15) | 194 | 235 |  | 0.80 (0.66-0.96) |
| Stomach (C16) | 252 | 242 |  | 1.00 (0.84-1.19) |
| Small intestinal (C17) | 31 | 36 |  | 0.83 (0.51-1.34) |
| Colon (C18) | 985 | 1,024 |  | 0.92 (0.84-1.01) |
| Rectum excluding anus (C20) | 607 | 625 |  | 0.93 (0.83-1.04) |
| Liver (C22) | 102 | 164 |  | 0.60 (0.47-0.77) |
| Pancreas (C25) | 309 | 326 |  | 0.91 (0.78-1.06) |
| Respiratory system (C30-C39) | 1,753 | 1,765 |  | 0.94 (0.88-1.01) |
| Larynx (C32) | 115 | 142 |  | 0.77 (0.60-0.99) |
| Lung (C34) | 1,591 | 1,582 |  | 0.95 (0.89-1.02) |
| Melanoma (C43) | 505 | 442 |  | 1.09 (0.96-1.24) |
| Breast (C50) – women only | 170 | 176 |  | 0.96 (0.78-1.18) |
| Female genital organs (C51-C58) | 59 | 62 |  | 0.93 (0.65-1.33) |
| Cervix (C53) | 10 | 10 |  | 0.92 (0.38-2.21) |
| Uterus (C54-C55) | 24 | 30 |  | 0.78 (0.45-1.33) |
| Ovarian (C56) | 16 | 19 |  | 0.85 (0.44-1.66) |
| Male genital organs (C60-C63) | 3,516 | 2,990 |  | 1.12 (1.07-1.18) |
| Prostate (C61) | 3,404 | 2,890 |  | 1.12 (1.07-1.18) |
| Testis (C62) | 81 | 71 |  | 1.12 (0.81-1.54) |
| Urinary system (C64-C68) | 789 | 781 |  | 0.97 (0.88-1.07) |
| Kidney (C64-C65) | 339 | 271 |  | 1.19 (1.01-1.40) |
| Bladder (C67) | 442 | 492 |  | 0.86 (0.76-0.98) |
| Sarcomas (C40, C41, C45-C49) | 191 | 127 |  | 1.43 (1.14-1.79) |
| Eye, brain and CNS (C69-C72) | 181 | 166 |  | 1.04 (0.84-1.29) |
| Lymphatic/hematopoietic tissue (C81-C96) | 822 | 820 |  | 0.96 (0.87-1.05) |

(1) Hazard ratio from Cox regression model matched on age and sex and adjusted for Charlson comorbidity index, COPD, educational level, affiliation with the labour market, job with heavy work and calendar time (5 years periods). Numbers in parentheses are 95% confidence interval
