# Supplementary material for: Chronic inflammation following hernia repair and cancer risk: A nationwide study
Source: Surg Open Sci. 2025 Jun 25;27:88–93. doi: 10.1016/j.sopen.2025.06.004 (PMC12301763; doi:10.1016/j.sopen.2025.06.004)
Supplement: Supplementary Table 4 — Supplementary analyses comparing open versus laparoscopic mesh hernia repair with readmission stratified by incisional and ventral mesh hernia repair and cancer incidence, Denmark, 1996–2014. [file mmc4.docx]

**Supplementary Table 4.**

**Supplementary analyses comparing open versus laparoscopic mesh hernia repair with readmission stratified by incisional and ventral mesh hernia repair and cancer incidence, Denmark, 1996-2014**

| **Analysis** | |  | **Comparison** |  | **Cancer cases** | **Risk time** | **Persons** | **IR (1)** |  | **Adj HR (2)** |
| --- | --- | --- | --- | --- | --- | --- | --- | --- | --- | --- |
|  |  |  |  |  |  |  |  |  |  |  |
| **Inguinal hernia repair with mesh** | | | |  |  |  |  |  |  |  |
|  | Laparoscopic |  | Readmitted (3) |  | 74 | 3,738.6 | 860 | 1,979.4 |  | 1.44 (1.13-1.83) |
|  |  |  | Not readmitted |  | 933 | 71,923.5 | 13,112 | 1,297.2 |  | 1.00 (ref) |
|  |  |  |  |  |  |  |  |  |  |  |
|  | Open |  | Readmitted (3) |  | 592 | 33,051.8 | 4,983 | 1,791.1 |  | 1.18 (1.09-1.29) |
|  |  |  | Not readmitted |  | 9,663 | 741,351.8 | 87,387 | 1,303.4 |  | 1.00 (ref) |
|  |  |  |  |  |  |  |  |  |  |  |
| **Ventral hernia repair with mesh** | | | |  |  |  |  |  |  |  |
|  | Laparoscopic |  | Readmitted (3) |  | 53 | 3,180.8 | 722 | 1,666.3 |  | 1.11 (0.83-1.50) |
|  |  |  | Not readmitted |  | 330 | 24,267.8 | 5,195 | 1,359.8 |  | 1.00 (ref) |
|  |  |  |  |  |  |  |  |  |  |  |
|  | Open |  | Readmitted (3) |  | 139 | 8,125.5 | 1,319 | 1,710.7 |  | 1.17 (0.97-1.39) |
|  |  |  | Not readmitted |  | 949 | 68,773.3 | 9,673 | 1,379.9 |  | 1.00 (ref) |
|  |  |  |  |  |  |  |  |  |  |  |

(1) Incidence rate per 100,000 person-years.

(2) Hazard ratio from Cox regression model matched or adjusted on age and sex and adjusted for Charlson comorbidity index, COPD, educational level, affiliation with the labour market, job with heavy work and calendar time (5 years periods).
(3) Comparison of mesh hernia patients with and without readmission within 30 days of mesh operation.
